# [Retracted] Tanshinone‑IIA attenuates the deleterious effects of oxidative stress in osteoporosis through the NF‑κB signaling pathway

**DOI:** 10.3892/mmr.2023.13140

**Published:** 2023-12-05

**Authors:** Shaowen Zhu, Wanfu Wei, Zhiwei Liu, Yang Yang, Haobo Jia

Mol Med Rep 17: 6969–6976, 2018; DOI: 10.3892/mmr.2018.8741

Following the publication of this paper, it was drawn to the Editor's attention by a concerned reader that the images shown in Fig. 1E to represent the results from osteoclast differentation experiments were strikingly similar to data appearing in different form in another article written by different authors at different research institutes [Yang Y, Su Y, Wang D, Chen Y, Wu T, Li G, Sun X and Cui L: Tanshinol attenuates the deleterious effects of oxidative stress on osteoblastic differentiation via Wnt/FoxO3a signaling. Oxid Med Cell Longev 6: 351895, 2013].

Owing to the fact that the contentious data in the above article had already been published prior to its submission to *Molecular Medicine Reports*, the Editor has decided that this paper should be retracted from the Journal. The authors were asked for an explanation to account for these concerns, but the Editorial Office did not receive a reply. The Editor apologizes to the readership for any inconvenience caused.

